# Fasciola cinereum: a novel choke point for epilepsy treatment

**DOI:** 10.1186/s42494-024-00182-3

**Published:** 2025-01-08

**Authors:** Lilong Yu, Dongxiao Jiang, Yi Wang, Zhong Chen, Lin Yang

**Affiliations:** 1 Key Laboratory of Neuropharmacology and Translational Medicine of Zhejiang Province, The First Affiliated Hospital, School of Pharmaceutical Sciences, Zhejiang Chinese Medical University, Hangzhou, 310053 China; 2 Institute of Pharmacology & Toxicology, College of Pharmaceutical Sciences, School of Medicine, Zhejiang University, Hangzhou, 310053 China

**Keywords:** Fasciola cinereum, Seizure node, Epilepsy treatment

## Abstract

Ablation of seizure foci represents a crucial therapeutic approach for epilepsy. Traditionally, the seizure foci are predominantly located in the anterior hippocampus and amygdala. However, recent research by Ivan Soltesz and his colleagues described the posterior hippocampal fasciola cinereum (FC) as a region activated during seizures. Their findings demonstrate that inhibition and ablation of FC reduce seizures frequency. Therefore, FC emerges as a critical seizure node within the posterior hippocampus, playing an important role in epilepsy treatment.

## Background

Epilepsy affects over 70 million people worldwide, with only approximately 60% of epilepsy patients achieving seizure control through anti-seizure medications [1, 2]. Temporal lobe epilepsy (TLE) is the common type of epilepsy and frequently exhibits drug resistance. For patients with drug-resistance epilepsy, surgery and deep brain stimulation are alternative treatments. Prior to surgery, doctors usually use stereoelectroencephalography (sEEG) to identify the brain tissue responsible for initiating and propagating seizure activity. For the surgery of the TLE, electrodes are typically placed only in the anterior and central regions of the hippocampus, often ignoring the posterior hippocampus, where the seizure node may remain. This raises the question of whether a seizure node remains in the posterior hippocampus [3]. As a supporting evidence, previous research has identified no significant relationship between the preservation of the posterior hippocampal and seizure freedom [4]. However, the existence of the seizure node in posterior hippocampus remains unresolved. Recently, the research team led by Ivan Soltesz investigated the role of a specific nucleus, FC, a longitudinal midline structure in the posterior-hippocampus, as a key seizure node for epilepsy treatment in both animal models and epilepsy patients [5].

## Main text

FC is a novel nucleus, and only a few anatomical studies have been conducted. The function of FC is largely unknown, with only one previous study suggesting its important role in acquisition of visual contextual memory [6]. First, the role of FC neurons in epilepsy was investigated by using the single-chain fast light and activity-regulated expression (scFLARE) [7], a tool specifically designed for labeling neurons corresponding to active events. Upon neurons activation, high calcium activates TEV protease, and light changes the conformation of hybrid light-oxygenvoltage-sensing domain (hLOV), exposing Tobacco Etch Virus protease cleavage site (TEVcs) for cleavage. The transcription factor (TF) is subsequently released, translocated to the nucleus, and drives the expression of the selected reporter gene. Compared with traditional c-fos^+^ staining, scFLARE offers greater specificity for short event within a limited time window, such as seizure event that usually last only a few minutes. Data from scFLARE showed that Purkinje cells in the FC are involved in epileptic seizures. Second, the authors monitored epileptic activity in the FC neurons with two-photon calcium imaging and verified that Purkinje cells in the FC were activated following convulsive seizures. To further verify the causal role of FC neurons in epilepsy, the authors used PCP4-Cre transgenic mouse line with a Cre-recombinase–dependent virus (AAV-Syn-SIO-stGtACR2-FusionRed) to inhibit the Purkinje cells in the FC, and found that this greatly reduced the duration of epileptic seizures.

To further address whether FC is critically involved in human seizure activity, the author recorded sEEG from the brain regions (frontal, temporal, insular, occipital, and thalamic) of six patients to identify the source of the seizures and inform future surgical interventions. Interestingly, epileptiform discharges were recorded in FC nucleus in all six patients. For one of the patients who underwent laser interstitial thermal therapy, the frequency of epilepsy decreased from 3–4 times per month to 2 times per month, although complete seizure freedom was not achieved. However, following a second ablation targeting the posterior hippocampal tail, the frequency of epilepsy was reduced by 83%. The above results indicate that FC nucleus is a critical seizure node in the posterior hippocampus in epilepsy patients.

**Fig. 1:**
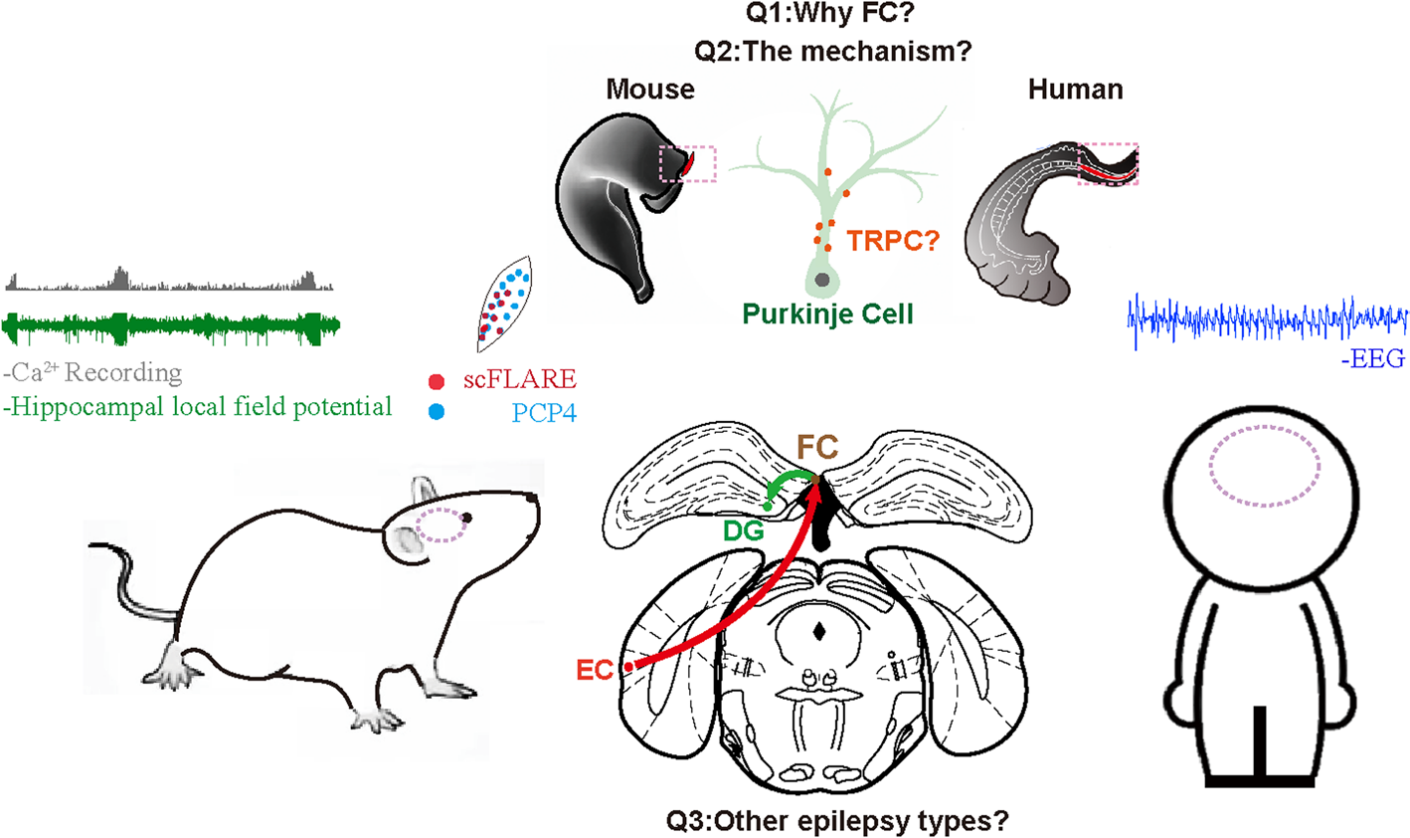
FC is involved in epileptic activity in animals and humans, but several points remain further investigation

In summary, the authors found that, apart from the anterior hippocampal and amygdala foci mentioned in previous studies, there is another key seizure point: FC. When a seizure node is detected and excised, the FC deserves attention. This research provides a new direction for the clinical treatment of epilepsy. For further investigation of FC, there are still several points worth considering (Fig. 1): (1) Apart from FC, other nuclei in the posterior hippocampus involved in the epileptic seizures of TLE deserve further investigation.  The indusium griseum (IG) is close to the FC and is considered to be a vestige of the hippocampus or an extension of the dentate gyrus via the FC. Gene sequencing [8] and protein expression [9] have revealed similarities between IG and FC. These above regions present promising avenues for future research. (2) It's worth investigating how the FC becomes a seizure node and the role of FC in initiation, propagation, or termination process of seizures. Previous studies have reported that the canonical transient receptor potential (TRPC) non-selective cation channels are maximally expressed in cerebellar Purkinje neurons. TRPC is sustainedly activated in pathological conditions such as epilepsy. Dysregulation of calcium (Ca^2+^) homeostasis leads to progressive disruption of the Purkinje cell dendrites and exacerbates excitotoxicity-based secondary brain injury expansion, which is a key driver of epilepsy [10]. Importantly, TRPC3 is also expressed in the FC, suggesting that epileptic seizures may damage Purkinje cells, thus causing FC to become a seizure node and aggravating the severity of epilepsy. Further studies could explore therapeutic strategies based on this mechanism. (3) Regarding connectivity, FC receives input from the lateral entorhinal cortex and sends output to the crest of the dentate gyrus. It is also worth elucidating the upstream and downstream circuits of FC in both physiological conditions and pathological states, such as epilepsy. This research confirmed that in patients with non-TLE, the FC is also involved in epileptic activities. Therefore, it is important for further clinical application to identify non-TLE patients and determine whether their FC is a seizure node. For safety considerations, whether FC ablation causes any side effect needs long-term monitoring. Overall, this study paves the way for the FC as a choke point for seizure in TLE and highlights it as a promising target for the future treatment of epilepsy.

## Conclusions

In conclusion, this study highlighted FC as a novel choke point for epilepsy treatment. Future efforts should focus on exploring other nucleus in the posterior-hippocampus and investigating the mechanism of FC becoming a seizure node.

## Data Availability

Not applicable.

## Abbreviations

FC: Fasciola cinereum
scFLARE: Single-chain fast light and activity-regulated expression
TLE: Temporal lobe epilepsy
TRPC: Canonical transient receptor potential

## References

[1] Wang Y, Chen Z. An update for epilepsy research and antiepileptic drug development: Toward precise circuit therapy. Pharmacol Ther. 2019;201:77–93. 31128154 10.1016/j.pharmthera.2019.05.010

[2] Thijs RD, Surges R, O’Brien TJ, Sander JW. Epilepsy in adults. Lancet. 2019;393(10172):689–701. 30686584 10.1016/S0140-6736(18)32596-0

[3] Schramm J. Temporal lobe epilepsy surgery and the quest for optimal extent of resection: a review. Epilepsia. 2008;49(8):1296–307. 18410360 10.1111/j.1528-1167.2008.01604.x

[4] Dasgupta D, Finn R, Chari A, Giampiccolo D, de Tisi J, O’Keeffe AG, et al. Hippocampal resection in temporal lobe epilepsy: Do we need to resect the tail? Epilepsy Res. 2023;190:107086. 36709527 10.1016/j.eplepsyres.2023.107086 PMC10626579

[5] Jamiolkowski RM, Nguyen QA, Farrell JS, McGinn RJ, Hartmann DA, Nirschl JJ, et al. The fasciola cinereum of the hippocampal tail as an interventional target in epilepsy. Nat Med. 2024;30(5):1292–9. 38632391 10.1038/s41591-024-02924-9 PMC11108783

[6] Park SB, Lim HY, Lee EY, Yoo SW, Jung HS, Lee E, et al. The fasciola cinereum subregion of the hippocampus is important for the acquisition of visual contextual memory. Prog Neurobiol. 2022;210:102217. 34999186 10.1016/j.pneurobio.2022.102217

[7] Sanchez MI, Nguyen QA, Wang W, Soltesz I, Ting AY. Transcriptional readout of neuronal activity via an engineered Ca(2+)-activated protease. Proc Natl Acad Sci U S A. 2020;117(52):33186–96. 33323488 10.1073/pnas.2006521117 PMC7777206

[8] Shi H, He Y, Zhou Y, Huang J, Maher K, Wang B, et al. Spatial atlas of the mouse central nervous system at molecular resolution. Nature. 2023;622(7983):552–61. 37758947 10.1038/s41586-023-06569-5 PMC10709140

[9] Sanders M, Petrasch-Parwez E, Habbes HW, Düring MV, Förster E. Postnatal developmental expression profile classifies the indusium griseum as a distinct subfield of the hippocampal formation. Front Cell Dev Biol. 2020;8:615571. 33511122 10.3389/fcell.2020.615571 PMC7835525

[10] Parmar J, von Jonquieres G, Gorlamandala N, Chung B, Craig AJ, Pinyon JL, et al. TRPC Channels Activated by G Protein-Coupled Receptors Drive Ca(2+) Dysregulation Leading to Secondary Brain Injury in the Mouse Model. Transl Stroke Res. 2024;15(4):844–58. 37462831 10.1007/s12975-023-01173-1 PMC11226524

